# Strength and Biocompatibility of Heparin-Based Calcium Phosphate Cement Grafted with Ferulic Acid

**DOI:** 10.3390/polym13132219

**Published:** 2021-07-05

**Authors:** Kai-Chi Chang, Jian-Chih Chen, I-Tse Cheng, Ssu-Meng Haung, Shih-Ming Liu, Chia-Ling Ko, Ying-Sui Sun, Chi-Jen Shih, Wen-Cheng Chen

**Affiliations:** 1 Advanced Medical Devices and Composites Laboratory, Department of Fiber and Composite Materials, Feng Chia University, Taichung 407, Taiwan; ketty60221@gmail.com (K.-C.C.); yaschen@fcu.edu.tw (I.-T.C.); dream161619192020@gmail.com (S.-M.H.); 0203home@gmail.com (S.-M.L.); rayko1024.rb@gmail.com (C.-L.K.); 2 Department of Orthopedics, College of Medicine, Kaohsiung Medical University, Kaohsiung 807, Taiwan; d830191@gmail.com; 3 Department of Orthopedics, Kaohsiung Medical University Hospital, Kaohsiung 807, Taiwan; 4 School of Dental Technology, College of Oral Medicine, Taipei Medical University, Taipei 110, Taiwan; yingsuisun@tmu.edu.tw; 5 Department of Fragrance and Cosmetic Science, College of Pharmacy, Kaohsiung Medical University, Kaohsiung 807, Taiwan; 6 Drug Development and Value Creation Research Center, Kaohsiung Medical University, Kaohsiung 807, Taiwan; 7 Department of Medical Research, Kaohsiung Medical University Hospital, Kaohsiung 807, Taiwan; 8 Dental Medical Devices and Materials Research Center, College of Dental Medicine, Kaohsiung Medical University, Kaohsiung 807, Taiwan

**Keywords:** apatite, nanorods, templates, calcium phosphate bone cement, biocompatibility, mineralization

## Abstract

The biomimetic synthesis of carbonated apatites by biomolecule-based templates is a promising way for broadening apatite applications in bone tissue regeneration. In this work, heparin was used as an organic template to prepare uniform carbonate-based apatite nanorods (CHA) and graft ferulic acid (F-CHA) for enhanced bone mineralization. Next, by combining calcium phosphate cement (CPC) with different F-CHA/CPC ratios, a new type of injectable bone cement combined with F-CHA bioactive apatite was developed (CPC + F-CHA). The physicochemical properties, biocompatibility, and mineralization potential of the CPC + F-CHA composites were determined in vitro. The experimental results confirmed the preparation of highly biocompatible CHA and the compatibility of F-CHA with CPC. Although CPC + F-CHA composites with F-CHA (2.5 wt%, 5 wt%, and 10 wt%) showed a significant reduction in compressive strength (CS), compositing CPC with 10 wt% F-CHA yielded a CS suitable for orthopedic repair (CS still larger than 30 MPa). Spectroscopic and phase analyses revealed that the phase of the hydrothermally synthesized CHA product was not modified by the heparin template. Injection and disintegration tests indicated that the CPC + F-CHA composites have good biocompatibility even at 10 wt% F-CHA. D1 osteoprogenitor cells were cultured with the composites for 7 days in vitro, and the CPC + 10%F-CHA group demonstrated significantly promoted cell mineralization compared with other groups. Given these results, the use of over 10% F-CHA in CPC composites should be avoided if the latter is to be applied to load-bearing areas. A stress-shielding device may also be recommended to stabilize these areas. These newly developed biocompatible CPC + F-CHA have great potential as osteoconductive bone fillers for bone tissue engineering.

## 1. Introduction

The composition, structure, and particle size of artificially synthesized carbonate-based apatite nanorods (CHA) are similar to those of natural bone. Moreover, CHA shows good biological activity and has an important influence on bone mineralization [1,2,3]. The Ca^2+^ and PO_4_^3−^ in hydroxyapatite (HA) have the molecular formula Ca_10_(PO_4_)_6_(OH)_2_, which can be replaced by other ions, and the resulting materials can be used in medical devices. For example, the OH^−^ and PO_4_^3−^ in apatite can be replaced with CO_3_^2−^ to obtain a material with a composition and structure similar to those of bone apatite. The chemical formula of CHA is Ca_10−_*_x_*(PO_4_)_6−_*_x_*(CO_3_)*_x_*(OH)_2−_*_x_* (0 ≤ *x* ≤ 2) [3].

The preparation of nano-structured apatite is challenged by issues such as large apatite size distributions, uneven geometric shape distribution, and powder agglomeration during synthesis [3,4,5,6,7,8]. Therefore, several scholars have sought to determine how to control the growth of apatite crystals precisely and adjust the characteristics of the obtained material to achieve improved mechanical properties and biocompatibility in vivo and in vitro [4]. According to the literature [5], natural glycosaminoglycans, such as heparin and chondroitin sulfate, may be used as templates to control crystal growth. Making the natural organic template as the possible precursor of apatite more worthy of in-depth discussion and research [6]. Besides using CHA to increase the rate of bone formation, some researchers have also used the template to homogenize the formation of nanometer-scale apatite, which can be employed to carry drugs to target sites or achieve the slow and sustained release of drugs [7]. Because of the wide applications of CHA, especially CHA nanorods, in the biomedical field, the material has continued to receive extensive research attention [3,8].

Ferulic acid is a stable water-soluble endothelin receptor antagonist. The phenolic hydroxyl group of ferulic acid can reduce oxygen free radicals, thereby reducing damage to cells and tissues. As such, it has gradually attracted the attention of scientists [9,10]. Studies have found that, in addition to anti-oxidation and free-radical scavenging effects, ferulic acid also has anti-inflammatory, anti-platelet aggregation, anti-apoptosis, and anti-DNA damage properties [9]. Ferulic acid can effectively increase osteopontin after bone matrix mineralization, increase bone growth factors, such as VEGF, TGF-β, and BMP, and promote local vascular proliferation. Furthermore, it can enhance the activity of osteoblasts and lead to the proliferation, differentiation, and mineralization of these cells. Folwarczna et al. found that ferulic acid participates in the production of osteoblasts through dishevelled (Dsh) and β-catenin via the Wnt signaling pathway. β-Catenin controls the main regulator of osteoblasts (i.e., Runx2), promotes osteoblast differentiation, and leads to bone formation [10].

Calcium phosphate bone cement (CPC) is a biocompatible material with fast-curing and self-hardening properties. Therefore, CPC has been widely used in orthopedics and facial bone repair for many years. The diversity of CPCs could be expanded by mixing different types of calcium phosphate with a hardening liquid [11,12,13]. After the hardening reaction, the main product phases of CPC are apatites, dicalcium phosphate dihydrate (DCPD), and amorphous calcium phosphates (ACP); the products can present as single-phase or multi-phase calcium phosphates. Recent research has attempted to improve the properties of CPCs by endowing them with antibacterial or drug-release properties [14,15]. Functionalized CPC may revolutionize the science of bone repair. CHA nanorods added to the CPC matrix may serve as initial core sites to accelerate the hardening reaction; therefore, nano-sized reinforcements can improve the crystallinity of the CPC after the hardening reaction [16]. The mechanical properties of CPC, especially the osseointegration required for load-bearing applications, can effectively be improved if the appropriate amount of nanoparticles is added to the cement.

In the present study, biomimetically synthesized heparin, an organic template that could regulate the growth of apatite nanorods, was used to react Ca^2+^ and PO_4_^3−^ ions with atmospheric CO_2_ [3,17]. Uniform nanorods of CHA with heparin were then used as a mediator to graft ferulic acid to form a ferulic acid-based drug template carrier (F-CHA). Next, the carrier was combined with a CPC matrix to form a series of CPC + F-CHA composites. The physical, chemical and mechanical properties of the composites were studied, and the biocompatibility and mineralization potential of the CPC + F-CHA drug-impregnated composites was assessed by in vitro cell culture with D1 osteoprogenitor cells. The results of this work provide a preliminary assessment of the future in vivo applications of the developed CPC + F-CHA composites.

## 2. Materials and Methods

### 2.1. Materials

The raw materials used to prepare the CHA nanorods included calcium nitrate (Ca(NO_3_)_2_, Katayama Chemical Industries Co., Ltd., Osaka, Japan) and diammonium hydrogen phosphate ((NH_4_)_2_HPO_4_, HSE Pure Chemicals, Calgary, AB, Canada). Sodium hydroxide (NaOH, Shimakyu Pure Chemicals, Osaka, Japan) was used to adjust the solution pH. Heparin (pharmaceutical-grade, Heparin Leo, medical injection for intravenous administration, 25,000–100,000 IU/mL) was used as a template to regulate the orientation of CHA and enable the grafting of ferulic acid (Sigma-Aldrich^®^, St Louis, MO, USA) to endow the resultant CHA nanorods with unique functionality. Nondispersive CPC was prepared from tetracalcium phosphate (TTCP)/dicalcium phosphate anhydrous (DCPA) as previously described [18,19]. TTCP powder with a mean particle size of 10 μm was prepared from the reaction of Ca_2_P_2_O_7_ (Alfa Aesar, Johnson Matthey Company, Devens, MA, USA) and CaCO_3_ (Shimakyu Pure Chemicals, Osaka, Japan). DCPA (CaHPO_4_, Acros Organics, Geel, Belgium) with a particle size distribution of 1–3 μm and a purity of 98% was also used.

The cell culture chemicals used in this work included α-minimum essential medium (α-MEM; Gibco^®^, Thermo Fisher Scientific Inc., Waltham, MA, USA), penicillin/streptomycin (Biological Industries, Kibbutz Beit Haemek, Israel), horse serum (Biolegend Co., San Diego, CA, USA), sodium bicarbonate (Sigma-Aldrich, St Louis, MO, USA), trypsin (Gibco^®^, Thermo Fisher Scientific Inc., Waltham, MA, USA), and dimethyl sulfoxide (DMSO; Sigma-Aldrich, St Louis, MO, USA). An in vitro toxicology assay kit (XTT, Biological Industries, Kibbutz Beit Haemek, Israel) was also used.

### 2.2. Preparation of CHA, F-CHA, and CPC + CHA Composites

CHA nanorods were prepared according to our previously published paper [17]. Briefly, Ca(NO_3_)_2_ was mixed with 5, 10, or 20 g of heparin in 100 mL of aqueous solution and titrated with a solution containing (NH_4_)_2_HPO_4_. After titration, the final Ca/P atomic ratio in the precipitate system was a stoichiometric HA value of 1.67. The suspension was adjusted to pH 10, heat-inactivated at 95 °C for 4 h to promote CHA growth, washed, dried at 60 °C for 24 h as CHA with heparin, and then stored in a dry box to prevent the effects of humidity before further characterization. A transmission electron microscope (TEM, JEOL JEM-2100, JEOL, Tokyo, Japan) was used to examine the powders.

The conditions of F-CHA preparation were as follows. Exactly 0.005 g of ferulic acid was dispersed in 20 mL of double-distilled water (ddH_2_O), mixed uniformly, and then added to 1 g of CHA with heparin. The solution was stirred at 25 °C for 24 h, dried at 40 °C for 24 h as F-CHA, and then stored in a dry box.

Various CPC + F-CHA samples were prepared by uniformly mixing CPC powders with different weight percentages (2.5 wt%, 5 wt%, and 10 wt%) of F-CHA. Hardening solution was added to the above mixture at a liquid-to-powder ratio of 0.6 mL/g. The properties of the CPC and CPC + F-CHA samples were then compared [18,19].

### 2.3. Strength, Injection, and Dispersion Tests

The CPC composite pastes were filled into a stainless steel mold to form cylindrical specimens measuring 12 mm in height and 6 mm in diameter for the compressive strength (CS) test, and disc specimens measuring 3 mm in thickness and 6 mm in diameter were prepared for a diametral tensile strength (DTS) and all other cell culture tests. The CS test was conducted following ASTM F 451-99a, and the DTS test was performed following the procedures in [19] by using a desktop universal mechanical tester (LS 500, LLOYD Instruments, Tokyo, Japan) at a crosshead speed of 1 mm/min. The immersion ratio was set to 1 g of specimen/10 mL of Tris-buffer saline (TBS), and the specimens were immersed at 37 °C for 24 h before measurement. The fracture surfaces of the specimens were examined using a field emission scanning electron microscope (SEM, Hitachi S-3000 N, Hitachi, Tokyo, Japan). The specimens obtained after strength testing were ground for X-ray diffraction (XRD) characterization. An X-ray diffractometer (Shimadzu XRD-6000, Tokyo, Japan) with Ni-filtered Cu Kα radiation operated at 40 kV and 30 mA at a scanning speed of 2°/min was used.

The injectability and dispersion properties of the CPC + F-CHA composites were tested by injecting the pastes into a large amount of ddH_2_O at 37 °C. Here, the CPC + F-CHA composite was mixed thoroughly with hardening solution for 1 min and then filled into a needle-free 3 mL syringe within 2 min after starting to mix the pastes. The pastes were then injected into ddH_2_O. The dispersion of the pastes after injection into ddH_2_O at different observation times was measured. Table 1 shows the total synthetic component ratios and nomenclature of different groups prepared in this study.

### 2.4. In Vitro Measurements

#### 2.4.1. Cytotoxicity toward L929 Cells

The L929 cell line from newborn mouse fibroblasts was provided by the National Institute of Health in Taiwan and used for the cytotoxicity tests. Sample extracts were prepared at a sample-to-medium ratio of 1 g/5 mL. Here, the prepared samples were immersed in the culture medium for 24 h, and the upper layer of the liquid was collected as the extract. Cell viability was determined by culturing the L929 cells in the extracts, and cytocompatibility was determined according to ISO 10993-5:2009. Cells were seeded in a 96-well culture plate in α-MEM containing 10% horse serum culture medium at a density of 1 × 10^4^ cells/well and then cultured overnight. The culture medium was removed, and the extract was added to the L929 cell culture for 24 h. The used cell culture medium was aspirated, and 100 µL of new cell culture medium was added to the plates. The extract was aspirated, and afterward, tetrazolium salt (XTT cell proliferation kit; Biological Industries, Israel) was added to 50 μL/well and 100 μL/well culture medium. The assay is based on the ability of metabolically active cells to reduce the tetrazolium salt XTT to orange-colored compounds of formazan. The intensity of the dye is proportional to the number of metabolically active cells. The plate was incubated in a 5% CO_2_ incubator at 37 °C for 4 h; basically, 2 to 5 h is usually sufficient according to the instructions. After culture, the viability of the L929 cells was determined at OD_492_. The morphological characteristics of the cells were observed under an inverted optical microscope (IX71, Yuan Li Instrument Co., Ltd., Taipei, Taiwan).

#### 2.4.2. Attachment, Proliferation, and Mineralization of D1 Osteoprogenitor Cell Cultures on Sample Surfaces

Progenitor bone cells (D1) from a bone marrow mesenchymal stem cell line cloned from Balb/C mice were purchased from the American Type Culture Collection. D1 cells were cultured in Dulbecco’s modified Eagle medium (DMEM) supplemented with 10% fetal bovine serum at 37 °C under a humidified 5% CO_2_ atmosphere. The cells were used before their eighth passage.

##### Cell Attachment and Morphological Observation

The DTS specimens were cultured with D1 cells at a concentration of 1 × 10^4^ cells/well. The specimens were placed in a 48-well plate, and culture was performed for 1 h, 1 day, or 2 days. The specimens were washed sequentially with PBS, fixed with a mixture of 2.5% glutaraldehyde and paraformaldehyde, and dehydrated in a gradient alcohol sequence. The specimen was plated with aluminum, and the morphology of the attached cells was observed by SEM.

##### Semi-Quantitative Detection of Alkaline Phosphatase (ALP) Activity

The ALP test was performed 1, 4, 7, 10, and 14 days after the initial seeding of 1 × 10^5^ D1 cells on the DTS sample surface of the composites. The culture medium was changed thrice a week. After incubation, the cultured sample was washed with PBS, mixed with a new cell culture medium, and alamarBlue proliferation assay kit to quantitatively measure cell proliferation. After incubation for 4 h, the absorbance of the solution was measured with an ELISA reader at OD_570_ and OD_595_. The production of ALP, an early marker of osteogenesis, was determined as follows. The *p*-nitrophenyl phosphatase and TBS in the *p*-nitrophenyl phosphatase kit were added to 20 mL of sterile water and mixed evenly. After incubation, the cells were washed with PBS once and then added to the prepared solution. The cells were incubated for 30 min, and the absorbance of the cells was measured with an ELISA reader at OD_405_. The absorbance obtained is proportional to the amount of ALP secreted by the cells.

### 2.5. Statistical Analysis

The results were analyzed using a two-sample *t*-test and an analysis of variance (ANOVA). SPSS Statistics version 20 was employed for all statistical analyses, and Tukey’s test was used for post hoc analysis. The two-sample *t*-test compares differences between the averages of two samples, and an ANOVA compares the averages of multiple groups via two different estimates of variance.

## 3. Results and Discussion

### 3.1. Identification of CHA

#### 3.1.1. XRD Phase Identification

**Figure 1:**
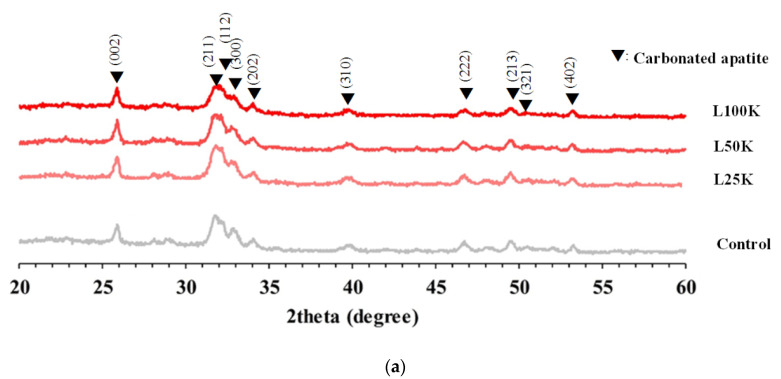

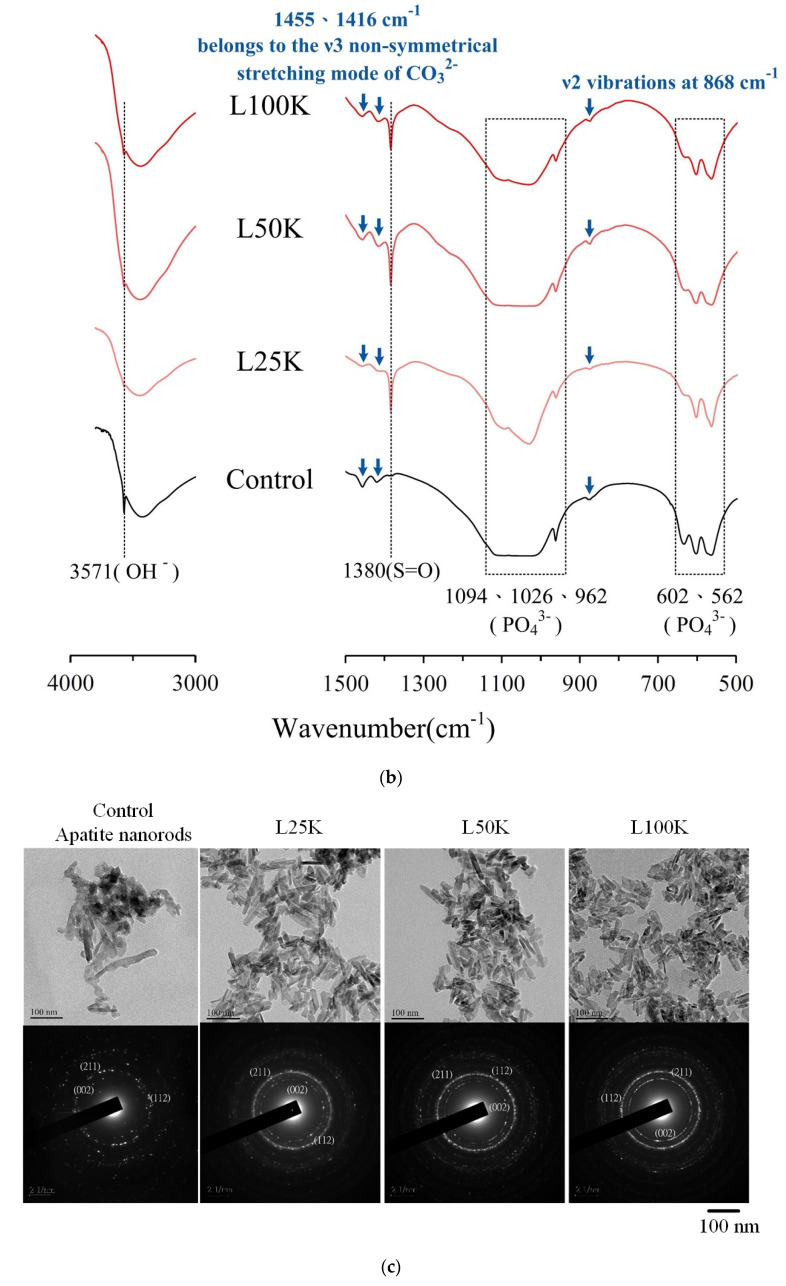
(**a**) XRD pattern of carbonated apatite nanorods templated with pharmaceutical heparin and prepared by hydrothermal synthesis. (**b**) The infrared absorption spectrum of carbonated apatite nanorods templated with pharmaceutical heparin and prepared by hydrothermal synthesis. (**c**) TEM image of carbonated apatite nanorods templated with pharmaceutical heparin and prepared by hydrothermal synthesis.

Comparison of the XRD patterns of the samples with the data of JCPDS No. 046-0905 revealed that the characteristic peaks of CHA correspond to those of apatite planes. This finding indicates that the addition of different concentrations of heparin does not change the hydrothermally synthesized CHA and that the processes described in this work can effectively prepare the CHA phase (Figure 1a). The relative intensities of diffraction peaks corresponding to (002), (211), (112), and (300) show a decreasing trend with the increase in heparin concentration, from L25K to L100k, it is presumed that the crystallinity and size decrease (Table 2).

#### 3.1.2. FTIR Spectral Analysis

The IR spectrum of the CHA product is shown in Figure 1b. The peak at 3571 cm^−1^ could be ascribed to the vibrations of the hydroxyl (OH^−^) groups, while the peaks at 1094 and 1026 cm^−1^ correspond to the asymmetric stretching modes of PO_4_^3−^. The band at 962 cm^−1^ is attributed to the symmetric stretching and bending modes of PO_4_^3−^ in CHA, and its formula is Ca_10−_*_x_*(PO_4_)_6−_*_x_*(CO_3_)*_x_*(OH)_2−_*_x_*, 0 ≤ *x* ≤ 2. The bands at 602 and 562 cm^−1^ represent the bending mode of PO_4_^3−^. These two functional groups are derived from the CHA phase [17,20,21,22]. The literature indicates that the absorption bands at 1416 and 1455 cm^−1^ belong to the ν_3_ non-symmetrical stretching mode of CO_3_^2−^, as well as ν_2_ vibrations at 868 cm^−1^. This may be due to the increase in carbon content and more substitution of carbonate in the crystal lattice. Thus, type B carbonate substitution occurs in HA [20,21]. The majority of calcium phosphate in the precipitation of the thermodynamic reaction in this study, HA, easily combines with CO_2_ in the atmosphere to form CHA. The results of these analyses demonstrate that the product of this experiment is type B CHA. In the IR spectrum of the heparin-grafted group, the peak at 1380 cm^−1^ reflects the asymmetric and symmetric O=S=O stretching vibrations of ester sulfate groups or the deformation vibrations of the C–H bond [17,22]. This finding confirms that heparin, a natural intermediate bridging agent, was indeed grafted on CHA and is involved in the regulation of CHA growth.

#### 3.1.3. TEM Images and CHA Nanorod Aspect Ratios

The TEM results in Figure 1c show that all analyzed groups have (002), (222), and (211) diffraction planes, which agrees with the XRD results. Thus, the synthesized product was confirmed to be CHA [2,23,24]. The shape of heparin-free nanorods is irregular and different in size; by comparison, the experimental groups with heparin as a template are shaped like nanorods. As the concentration of heparin increased, the CHA nanorods became thinner and shorter. Table 2 shows that the length of CHA nanorods between the non-template control and the L100K template group, as well as that between the L25K and L100K template groups, are significantly different (*p* < 0.05). Moreover, compared with those of the L50K and L100K groups, the diameter of CHA nanorods in the control group was significantly different (*p* < 0.05).

#### 3.1.4. Cytotoxicity of CHA

**Figure 2:**
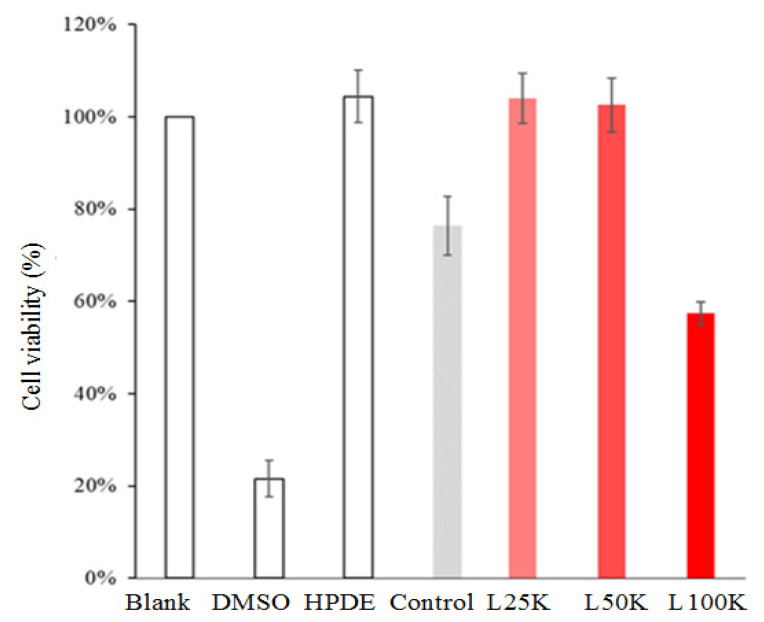
Viability of L929 cell cultures exposed to different extracts of CHA nanorods templated with different concentrations of pharmaceutical heparin (*n* = 6).

The biocompatibility test revealed that only the L100K group has cytotoxicity (Figure 2). The control, L25K, and L50K groups revealed no cytotoxicity. Since hydroxyapatite nanoparticles are formed through a combination of chemical synthesis pathways and cell dependence, cytotoxicity depends on their shape [3,25]. The analysis results showed that the biocompatibility of the L25K group is similar to other groups except for L100K, and no cytotoxicity was detected. Due to the heparin concentration playing an important factor in the chemical constitution and morphology, as well as biological properties of CHA, a higher concentration of heparin might lead to more CO_2_ absorption and thus lower the Ca/P ratio [21]. The morphology of CHA was small (Figure 1c) and the substitution of carbonate can enhance the solubility of CHA with a lower Ca/P ratio in a group of L100K compared to other groups of control CHA, L25K, and L50K. In addition, a large amount of soluble CHA with lower Ca/P tends to make the medium more acidic than CHA with higher Ca/P, resulting in poor biocompatibility. Among the groups tested, the L25K group demonstrated the most consistent morphology (i.e., the most uniform nanorods). Therefore, in subsequent experiments, L25K was compounded with CPC to evaluate the effect of F-CHA on the cell mineralization potential of D1 progenitor cells.

### 3.2. Identification of F-CHA and the Corresponding Templating Mechanism

**Figure 3:**
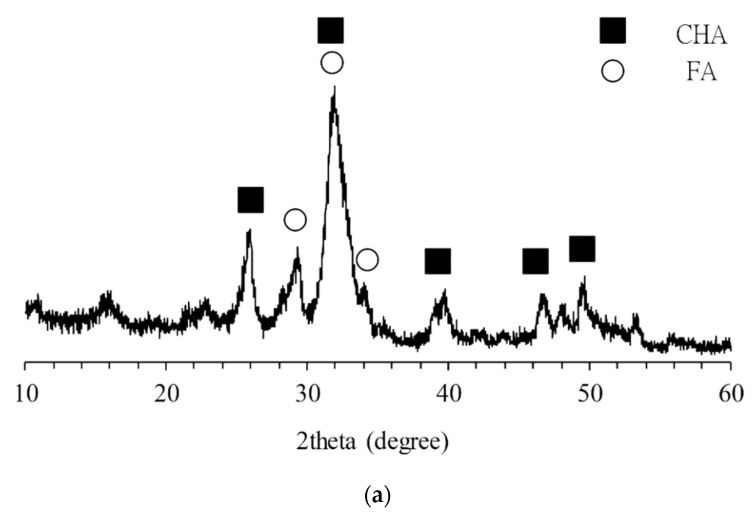

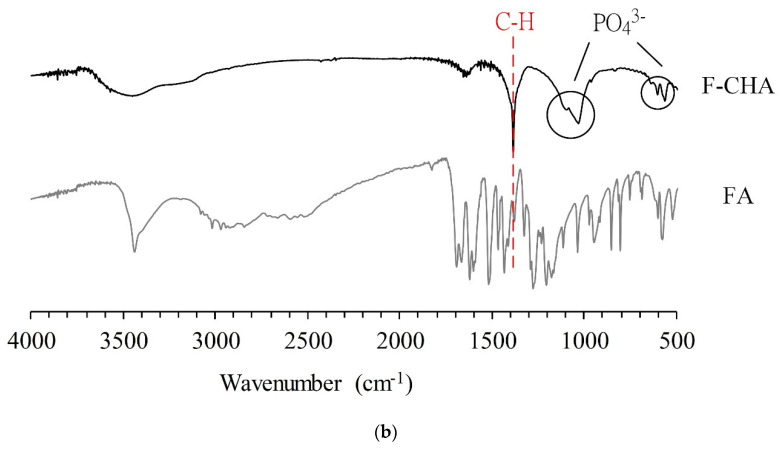
XRD pattern (**a**) and IR spectrum (**b**) of heparin-templated CHA nanorods impregnated with ferulic acid (F-CHA).

The XRD patterns of F-CHA showed an overlap of the characteristic peaks of CHA and FA. This finding indicates that ferulic acid was successfully impregnated into CHA (Figure 3a). FTIR spectral analysis of the impregnated ferulic acid showed that in addition to the stretching and bending modes of PO_4_^3−^ from CHA, a functional group different from CHA could be detected at 1384 cm^−1^. Comparison with the literature data [9] indicates that this band is the vibration peak of ferulic acid C-H (Figure 3b). The FTIR and XRD results are consistent and confirm that CHA was successfully impregnated with osteogenesis-promoting ferulic acid.

**Figure 4:**
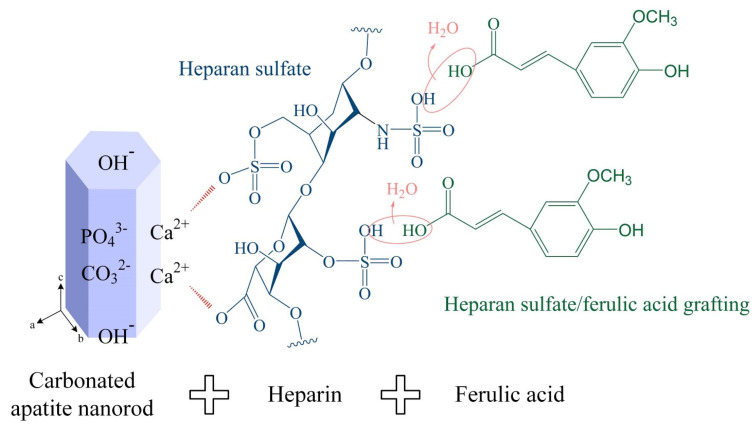
Possible grafting mechanism of polarized heparin-templated carbonated apatite nanorods grafted with ferulic acid.

In this study, heparin was used as an intermediate branching agent to regulate the growth of CHA nanorods and template to bind ferulic acid. The relevant mechanism may involve (1) the reaction of Ca^2+^ and PO_4_^3−^ with CO_2_ in a hydrothermal environment with heparin as a mediator to precipitate uniformly shaped CHA and (2) the use of the intermediate as a template to adsorb ferulic acid and promote the formation of F-CHA nanorods. A schematic of the possible reaction mechanism is shown in Figure 4.

Therefore, the carboxyl, carbonyl, and sulfate of the template heparin are thought to trigger the nucleation of apatite, thereby regulating CHA nucleation from the early stage. In the biomimetic manufacturing of apatite hybrid materials, the bi-molecular template is binding with ferulic acid through the polarized heparin template CHA, and the combination of molecules reduces the polarization charge of the original CHA. The interaction between CHA nanoparticles using heparin as a template and binding ferulic acid can improve this kind of nanoparticles with polarized charge-induced CHA aggregation (Figure 1c).

### 3.3. Release Characteristics, Strength, Phase, Morphology, and Injectability of CPC + F-CHA

#### 3.3.1. Individual Time and Cumulative Release Characteristics of Ferulic Acid

**Figure 5:**
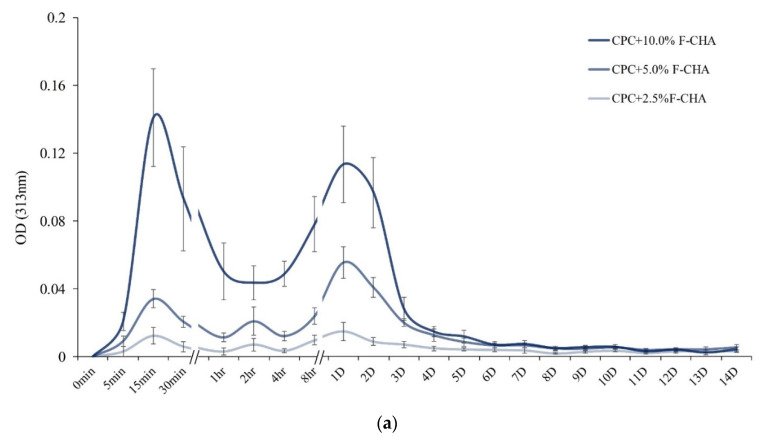

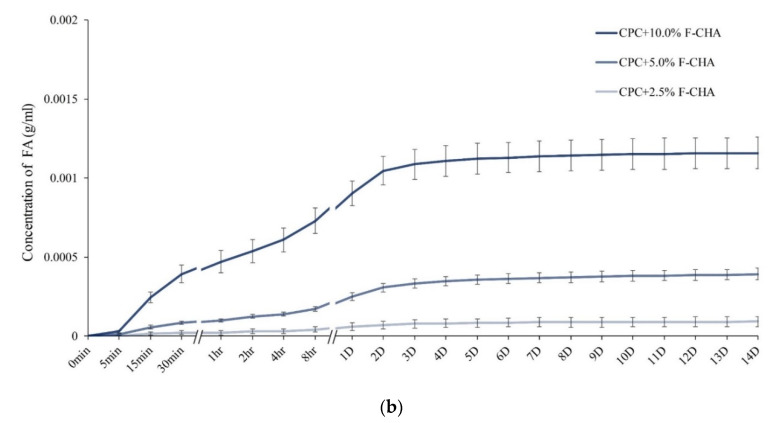
Individual time-release plots of ferulic acid (**a**) and cumulative release of carbonated apatite nanorods impregnated (**b**) with different concentrations of ferulic acid after immersion in ddH_2_O. The phenomenon of double-release peaks could be observed (*n* = 5).

The results of the drug release tests indicated that ferulic acid has a characteristic absorption peak at OD_313_ in the UV/Vis spectrum. Each group of CPC + F-CHA composites was immersed in deionized water for different times and then measured to determine the individual (Figure 5a) and cumulative (Figure 5b) release characteristics of ferulic acid. The individual release curves revealed two obvious release peaks for each group (Figure 5a). The first peak release occurred within 1 h and may be attributed to the initial burst release of ferulic acid on the surface of the CPC + F-CHA composites. The second gentle release peak was observed between 4 h and 3 days, which may be caused by the slow cumulative release of ferulic acid in the CPC composite. This phenomenon was confirmed by the cumulative release curves (Figure 5b). Ferulic acid was released in large quantities before 1 day and then demonstrated stable and sustained-release mode after 2 days. The results of these tests indicate that ferulic acid is released in three stages. The first stage is a burst early release that occurs within 1 h and is speculated to reflect the release of ferulic acid from the surface of the CPC + F-CHA composites. The second stage is an extensive release that occurs from 4 h to 3 days, which could be attributed to the diffused release of ferulic acid within the composites. Finally, the third stage is a slow-release that occurs after 3 days. These findings confirm that F-CHA may be an effective drug carrier.

#### 3.3.2. Compressive and Diametral Tensile Strengths of CPC + F-CHA Composites

**Figure 6:**
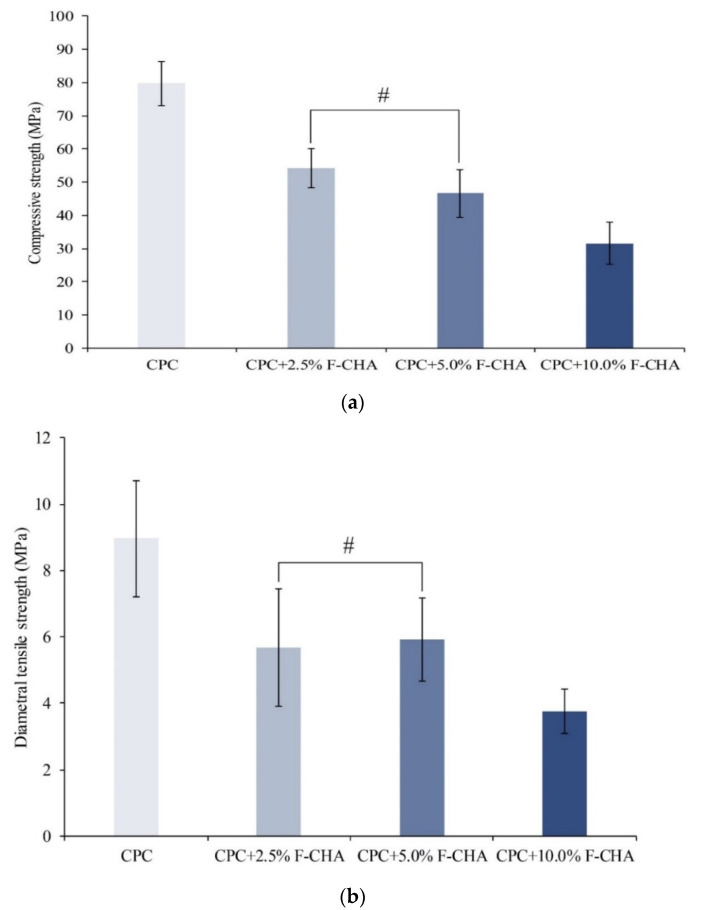
Compressive strength (**a**) and diametral tensile strength (**b**) of CPC composites with different ratios of ferulic acid-carbonated apatite nanorods after immersion in TBS for 1 day. Groups marked # indicate non-significant differences (*n* = 6; *p* > 0.05).

The strength of ideal bone implants should be comparable with that of human trabecular bone. The maximum CS of human trabecular bone is 30 MPa, and this standard can be used to evaluate whether experimental samples meet clinical needs [26]. The CS test results are shown in Figure 6a. The average CS of the CPC control after immersion in TBS for 1 day was 79 MPa. The addition of F-CHA revealed a decrease in CS. While no significant difference in the strengths of CPC + 2.5%F-CHA and CPC + 5.0%F-CHA was noted, the CS of the CPC + 10.0%F-CHA group was significantly poorer than that of these groups. The remaining average test value of CS was only 31 MPa. Figure 6b illustrates the DTS of each group of CPC + F-CHA composites. In general, the trend of the DTS of the composites was consistent with that of their CS.

Because the compositions of F-CHA and CPC are similar, the reason behind the remarkable decrease in strength of the composites after F-CHA reinforcement was studied. The addition of the reinforcement to the CPC matrix may lead to aggregation of the F-CHA nanorods on account of an increase in normal pressure. The formation of composite defects leads to stress concentration and a substantial decrease in strength. Therefore, CPC composites added with 10% F-CHA to CPC may be more suitable for non-stress-bearing bone defect repair than other clinical applications requiring high strength.

#### 3.3.3. SEM Images of the Fracture Surfaces and XRD Phase Identification of CPC + F-CHA Composites after Immersion for 1 Day

**Figure 7:**
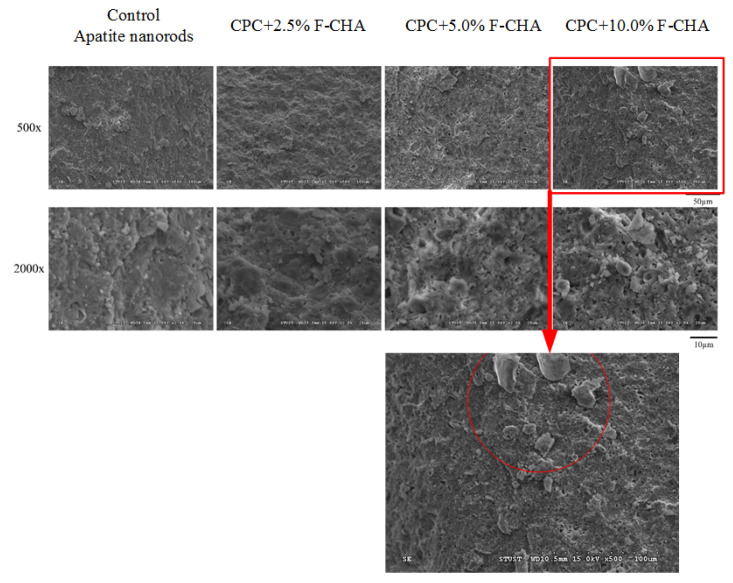
SEM images of fracture surfaces after compressive strength testing. CPC composites with different ratios of ferulic acid-carbonated apatite nanorods were measured after immersion in TBS for 1 day.

**Figure 8:**
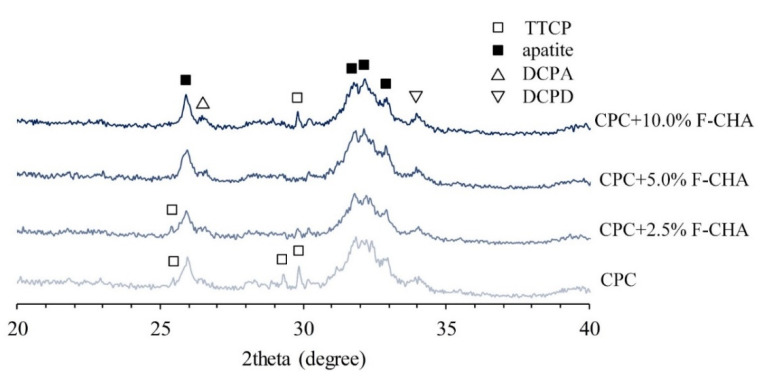
XRD phase identification of CPC composites with different ratios of ferulic acid-carbonated apatite nanorods after immersion in TBS for 1 day.

The CPC-only and CPC + F-CHA composites presented a coral reef-like structure after TBS immersion for 1 day (Figure 7). This structure is a type of apatite with an integrated surface. Each CPC + F-CHA composite immersed in TBS for 1 day was subjected to XRD analysis (Figure 8), and the results were compared with the standard files of JCPDS Nos. 09-0432, 77-0128, 72-0713, and 25-1137. The 2θ crystalline peaks of apatite are located at 25.90°, 31.76°, 32.19°, and 32.84°. The 2θ crystalline peak of DCPA occurs at 26.58°, and the crystalline peak of DCPD appears at 34.11°. These results show that, although the addition of F-CHA to CPC does not affect the formation of the apatite phase, the final CPC product is multi-phase, regardless of the presence or absence of F-CHA.

#### 3.3.4. Injection and Disintegration Tests of the CPC + F-CHA Composites in ddH_2_O

**Figure 9:**
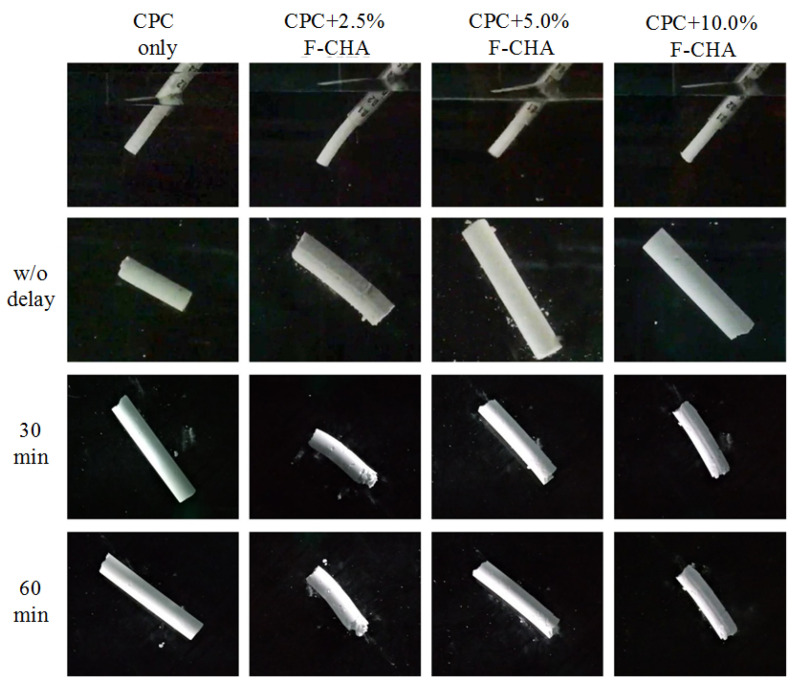
Injection and disintegration tests of CPC composites with different ratios of ferulic acid-carbonated apatite nanorods (CPC + F-CHA) after injection into ddH_2_O.

At present, CPC is defined as a combination of one or more calcium phosphates. When it is mixing with the hardening solution, it forms a slurry that can self-solidify or harden in situ at the bone defects. Therefore, one of the most important characteristics of CPC is that CPC can be formed in situ through a fluid-filled environment and still undergo a dissolution-precipitation hardening reaction. In this study, each group showed good injectability (Figure 9), and the CPC + F-CHA composites could be smoothly pushed out of the needle-free syringe without powder–liquid separation. After coming into contact with the solution for 1 h, the composite presented a cylindrical shape without disintegration, thereby indicating that it has good anti-dispersion ability. This finding indicates that if the composite is applied to clinical implantation in the future, the necessary repair could be completed even if body fluids or blood are encountered during the operation.

### 3.4. Cytotoxicity toward L929, Cell Attachment, Proliferation, and Differentiation of D1 Cultured with CPC + F-CHA

#### 3.4.1. Cell Viability and Morphologies of Sample Extract Culture with L929 Cells

**Figure 10:**
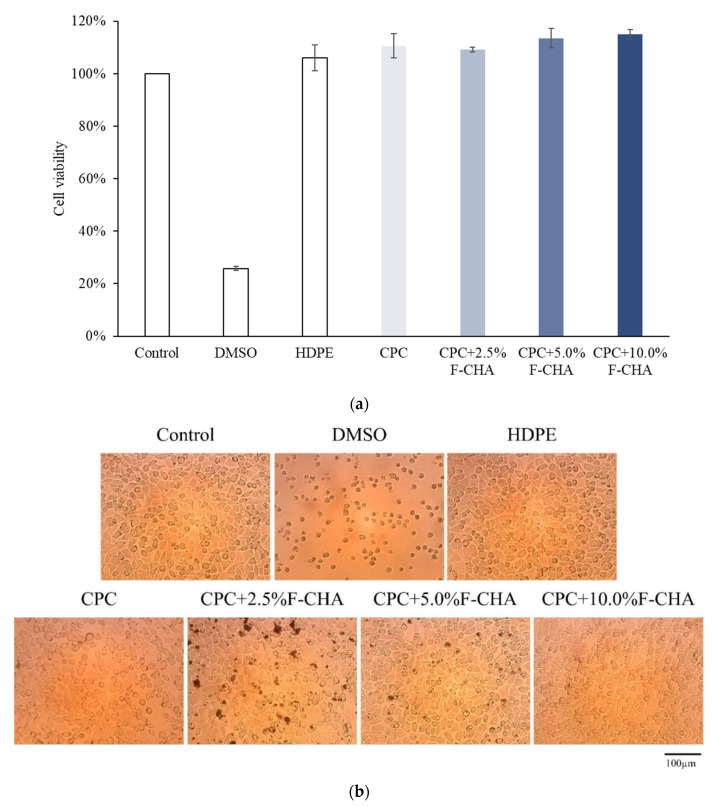
Quantitative cell viability (*n* = 6) (**a**) and qualitative cell morphologies (**b**) of L929 cells cultured for 24 h in solutions of CPC only and CPC composites with different ratios of ferulic acid-carbonated apatite nanorods (F-CHA).

According to the ISO-10993-5 standard, a > 30% reduction in cell viability indicates cytotoxic effects. The survival rate of cells cultured with the CPC + F-CHA composite extracts remained much higher than 70% regardless of the concentration of F-CHA that was added (Figure 10a), and the cell morphology of treated groups was similar to that of the control group (Figure 10b). Therefore, the CPC + F-CHA composites prepared in this work have no cytotoxicity.

#### 3.4.2. Morphology and ALP Activity of D1 Progenitor Bone Cells on Various CPC + F-CHA Composite Surfaces

**Figure 11:**
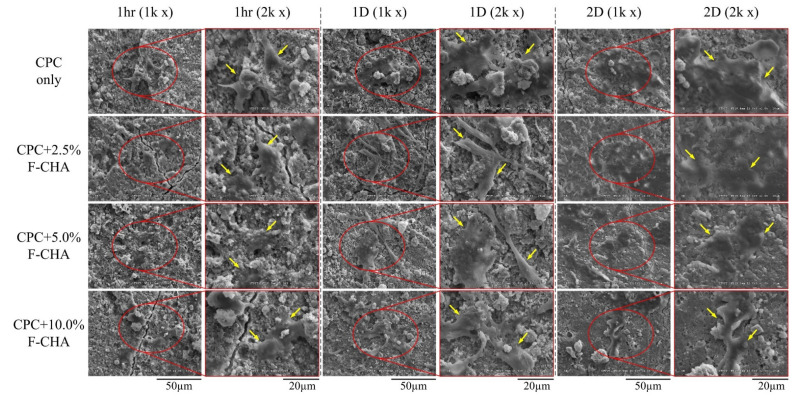
Morphologies of D1 progenitor bone cells cultured on the surfaces of CPC only and CPC composites with different ratios of ferulic acid-carbonated apatite nanorods for 1 h, 1 day, and 2 days.

D1 cells were exposed to the CPC + F-CHA composite materials for 1 h, 1 day, and 2 days, and their resulting morphology was assessed by SEM (Figure 11). D1 cells cultured on the surface of each group for 1 h initially adhered to the composite surface; by comparison, the cells in the control CPC-only group showed a relatively spherical shape. Cells that were not flat-attached to the surface of the CPC-only showed that cell affinity was worse than other CPC + F-CHA groups. Therefore, the addition of F-CHA could promote the attachment of bone cells. After culturing for 1 day, the cells on the surface of each PC + F-CHA composite have well adhered to the composite surface, and pseudopodia began to protrude from the culture. D1 cells cultured for 2 days on the composite revealed good adherence and began to proliferate.

**Figure 12:**
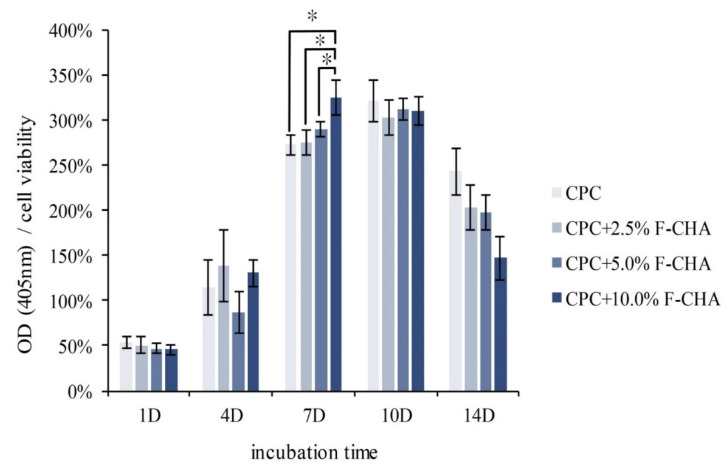
Semi-quantitative ALP activity of D1 cells seeded on CPC composites with different ratios of ferulic acid-carbonated apatite nanorods (CPC + F-CHA, *n* = 6). Groups marked * indicate statistically significant differences (*p* < 0.05).

An ALP test was conducted to confirm whether the addition of different amounts of F-CHA to the CPC-matrix affects the bone mineralization ability of D1 cells. ALP is a phosphatase produced by bone cells, and its structure is composed of over 500 amino acids [27]. The enzyme can assist in the hydrolysis of phosphate monoesters and release phosphate to participate in bone matrix mineralization [28]. Because D1 cells secrete large amounts of ALP before entering mineralization, ALP content can be used to determine whether D1 cells begin to mineralize. The amount of ALP produced was divided by the number of cells to assess the ability of a single D1 cell to produce ALP (Figure 12). The amount of ALP in each group peaked on day 7 of culture and was maintained until day 10, thereby indicating that the D1 cells began to mineralize on day 7. The quantity of ALP in the CPC + F-CHA composites did not change significantly. On day 7, D1 cells in the group containing CPC + 10%F-CHA began to secrete ALP at significantly higher levels compared with those in other groups. The results further showed that the higher the amount of F-CHA added, the greater the level of ALP secretion by D1 cells. Therefore, F-CHA does not affect the phase and biocompatibility of CPC, and the composite may be used as a carrier for sustained-release drugs to promote osteoprogenitor cell mineralization [9,29,30].

CPCs are osteoconductive because they attract the osteoprogenitor cells to attach, proliferate, migrate and express phenotypes, leading to the formation of new bone. Osteoconduction also depends on the compositions and the strategy of incorporating different types of biomolecules, drugs, or ions to promote CPC bone conductivity and absorption after clinical implantation. With some improvements in CPC composites, these in vitro tests can be used for preliminary screening. However, the most reliable assessment of osteoconduction and bone resorption is still the implantation of bone defects in the body. Ferulic acids are natural polyphenols present in various fruits and are the active ingredient in many herbal medicines that are known to have the ability to suppress the fusion and apoptosis of mature osteoclasts [29,31]. Therefore, ferulic acid exhibited protective effects against osteoclastic activity. Moreover, ferulic acid promoted the in vitro osteogenic differentiation of blood mesenchymal stem cells by inhibiting micro340 to induce *β*-catenin expression through hypoxia [29]. In addition, the study also showed that the proper immobilization of heparin into a polypyrrole matrix could promote mesenchymal stem cell differentiation towards osteoblast lineage [32], thus increasing the levels of ALP, indicating mineralization processes. We hypothesize that the inclusion of certain quantities of F-CHA to CPC will regulate the initial release of ferulic acid and combine with the release of heparin after a period of extension in vitro. In contrast, the biological activity of the released ferulic acid and heparin is maintained. The ability to stimulate localized osteogenesis at controlled rates through the release of ferulic acid and CHA with heparin from CPC could provide a potential therapeutic strategy.

## 4. Conclusions

The following conclusions may be drawn based on the results obtained in the present study:

1. L25K is a suitable mediator for impregnating bone, promoting ferulic acid, and compositing with CPC bone cement.
2. The release process of CPC + F-CHA composites shows three stages, including an early burst release from the composite surface, followed by the diffusion release of ferulic acid within the composites, and, finally, the slow release of the encapsulated drug. These findings prove that F-CHA may be an effective drug carrier.
3. The addition of F-CHA did not affect apatite formation. Increases in the addition of F-CHA resulted in a decrease in the strength of the product obtained. The CS of the CPC + 2.5, 5.0% F-CHA 2.5%, and 5% additive groups were approximately 50 MPa; thus, these materials may be used as load-bearing devices. The group added with CPC + 10%F-CHA achieved a CS of only 30 MPa; while this strength is relatively low, the obtained material may still find applications in non-load bearing orthopedic restoration.
4. After 1 h of culture, D1 cells cultured in the CPC-only group retained their spherical shape, but the cell morphology of cells in all other experimental CPC + F-CHA groups was flat. These results demonstrate that ferulic acid can promote progenitor bone cell attachment. On the 10th day of D1 cell culture, the amount of ALP production in the CPC + 10.0%F-CHA group was significantly higher than that in other treatment groups. This finding indicates that the addition of F-CHA in CPC increases the ability of a single cell to secrete ALP.
5. It can be seen that the controlled amount of heparin can prepare uniform nanorod-shaped CHA with good biocompatibility. The grafting of ferulic acid to CPC yielded a material that could be used as a slow-release drug carrier, thereby increasing the applicability of CPC in orthopedic tissue engineering.

## Figures and Tables

**Table 1:** Nomenclature of different groups prepared in this work. Pharmaceutical heparin was used as a template to prepare CHA nanorods and graft ferulic acid (F-CHA).

Designated Groups	Nomenclature
Comparative processes without heparin	Control CHA nanorods
Templated by heparin LEO INJ 25,000 unit/mL	L25K
Templated by heparin LEO INJ 50,000 unit/mL	L50K
Templated by heparin LEO INJ 100,000 unit/mL	L100K
Using L25K templated CHA for ferulic acid impregnation and composite CPC with a ratio of 2.5 wt% L25K/CPC	CPC + 2.5% F-CHA
Using L25K templated CHA for ferulic acid impregnation and composite CPC with a ratio of 5.0 wt% L25K/CPC	CPC + 5.0% F-CHA
Using L25K templated CHA for ferulic acid impregnation and composite CPC with a ratio of 10.0 wt% L25K/CPC	CPC + 10.0% F-CHA

**Table 2:** Average length, width, and the length-to-width ratio of carbonated apatite nanorods templated with different concentrations of pharmaceutical heparin (*n* = 30).

Dimensions and Aspect Ratio	Control CHA Nanorods Mean (S.D.) ^a^	L25K Mean (S.D.) ^a^	L50K Mean (S.D.) ^a^	L100K Mean (S.D.) ^a^
Length (nm)	70.08 (28.36)	70.32 (20.94)	60.62 (21.04)	52.76 (8.71)
Width (nm)	24.35 (6.16)	19.84 (5.20)	17.51 (3.69)	14.93 (6.16)
Aspect length-to-width ratio	2.88	3.54	3.46	3.53

^a^ S.D.: standard deviation.

## Data Availability

The data presented in this study are available on request from the corresponding author.
